# Physical properties of root cementum: Part 29. The effects of LED-mediated photobiomodulation on orthodontically induced root resorption and pain: a pilot split-mouth randomized controlled trial

**DOI:** 10.1093/ejo/cjac022

**Published:** 2022-05-11

**Authors:** John Sambevski, Alexandra K Papadopoulou, Matthew Foley, Kerem Dalci, Peter Petocz, Mehmet Ali Darendeliler, Oyku Dalci

**Affiliations:** Discipline of Orthodontics and Paediatric Dentistry, School of Dentistry, Faculty of Medicine and Health, The University of Sydney, Sydney Local Health District, NSW, Australia; Discipline of Orthodontics and Paediatric Dentistry, School of Dentistry, Faculty of Medicine and Health, The University of Sydney, Sydney Local Health District, NSW, Australia; Australian Centre for Microscopy and Microanalysis, The University of Sydney, NSW, Australia; Discipline of Orthodontics and Paediatric Dentistry, School of Dentistry, Faculty of Medicine and Health, The University of Sydney, Sydney Local Health District, NSW, Australia; Department of Statistics, Macquarie University, Sydney, NSW, Australia; Discipline of Orthodontics and Paediatric Dentistry, School of Dentistry, Faculty of Medicine and Health, The University of Sydney, Sydney Local Health District, NSW, Australia; Discipline of Orthodontics and Paediatric Dentistry, School of Dentistry, Faculty of Medicine and Health, The University of Sydney, Sydney Local Health District, NSW, Australia

## Abstract

**Objectives:**

To examine the effects of light-emitting diode (LED)-mediated photobiomodulation (PBM) on orthodontic root resorption and pain.

**Methods:**

Twenty patients (3 males, 17 females, mean age 15 years 6 months) needing bilateral maxillary first premolar extractions for orthodontic treatment were included in this single-centre, split-mouth randomized controlled trial. Both premolars received 150 g of buccal tipping force for 28 days. One side was randomly assigned to receive intraoral 850 nm wavelength, 60 mW/cm^2^ power, continuous LED illumination via OrthoPulse device (Biolux Research Ltd, Vancouver, British Columbia, Canada) for 5 minutes/day. The other side served as control. After 28 days, both premolars were extracted and scanned with micro-computed tomography for primary outcome assessment of root resorption crater volume measurements. For secondary outcome assessment, visual analogue scale pain questionnaires were used for both sides at 24 hours, 48 hours, 72 hours, and 7 days. Randomization was generated using www.randomization.com and allocation was concealed in sequentially numbered, opaque, sealed envelopes. Blinding was not possible during the experiment due to the use of tape to block light on control side of the devices. Assessors were blinded during outcome assessments.

**Results:**

All 40 premolars from 20 patients were included. There was no significant difference in the mean total root resorption between the LED PBM and control sides (mean 0.216 versus 0.284 mm^3^, respectively, *P* = 0.306). The LED side was associated with less pain at 24 hours (*P* = 0.023) and marginally more pain at subsequent time points, which was not statistically significant. No harms were observed.

**Limitations:**

Short study duration and the inability to blind patients and clinician during clinical part of study.

**Conclusion:**

This 28-day randomized split-mouth controlled trial showed that daily, LED-mediated PBM application, when applied for 5 minute/day, does not influence orthodontic root resorption. It is associated with significantly less pain 24 hours after the application of orthodontic force, but no difference thereafter. These results should be tested on patients undergoing a full course of orthodontic treatment.

**Trial registration:**

Clinical Trials Registry ACTRN12616000652471.

## Introduction

Orthodontically induced inflammatory root resorption (OIIRR) is an undesirable consequence of orthodontic tooth movement which is ubiquitous in orthodontic patients. Its aetiology is multifactorial and reported causes include individual genetic susceptibility and treatment-related considerations (1). Orthodontic tooth movement is a complex process which occurs through the remodelling of dental and periodontal tissues. This process requires biochemical changes in the periodontal ligament as well as the alveolar bone, two very different tissues with differing remodelling potentials (2). The pathogenesis of OIIRR is related to the biological processes of orthodontic tooth movement and both processes share localized acute inflammatory pathways involving common mediators.

There has been a growing interest in the field of accelerated tooth movement in the last few decades for various reasons. The reduction in treatment time is clearly desirable from both the patient and clinician’s standpoint as it could possibly decrease the risk of decalcification, dental caries, gingival inflammation, root resorption, and patient burn out (1). Techniques including local injection of cellular mediators, pulse electro-magnetic fields, mechanical vibration, surgically assisted orthodontics, low-level laser (LLL) therapy, and light-emitting diode (LED)-mediated photobiomodulation (PBM) have all been investigated as possible methods for increasing the rate of tooth movement (3). However, some of these modalities were reported to be inefficient, too invasive or impractical to incorporate into mainstream orthodontic practice.

PBM is a non-invasive biostimulatory technique and is the exposure to LLL or LED light. The term *biostimulation* was introduced in the 1960s to describe the photochemical interactions of low powered lasers with biological tissues and has since also been referred to as photostimulation, PBM, and photobiostimulation (4, 5). These terms are often used synonymously and encompass all forms of non-thermal light therapy. Both utilize low levels of red or near-infrared wavelengths ranging between 600 and 1000 nm, with 730 and 850 nm being considered the most appropriate for photo-biostimulatory effects (5). In orthodontics, human and animal studies have investigated the effects of PBM on mandibular growth enhancement, orthodontic pain, osseointegration of orthodontic miniscrews, accelerated tooth movement, and orthodontic root resorption (6–8). Most animal studies have shown that laser-mediated PBM increases the rate of orthodontic tooth movement due to accelerated bone remodelling via increased osteoblastic and osteoclastic cell proliferation and function (9). In humans, there are several studies that showed positive results on the potential role of LLLT PBM in accelerating orthodontic tooth movement, however others failed to show any significant effects (10–13).

Studies on the effect of LED-mediated PBM on orthodontic tooth movement and root resorption are more limited. While mostly favourable outcomes are suggested in animal studies (14–16), results of human studies on LED PBM and orthodontic root resorption are controversial as their study designs have several limitations such as not having a control group or subpar study protocol and assessment method (8, 17). There are no split-mouth, randomized controlled trials (RCTs) on humans investigating the role of LED-mediated PBM (LED PBM) to date. Furthermore, laser-mediated PBM has also shown the potential to reduce pain related to orthodontic adjustments (11) and although several studies exist on LLLT’s effects related to orthodontic pain, there are no RCTs using LED PBM for orthodontic adjustment pain. Therefore, the primary objective of the present study was to investigate the effects of LED-mediated PBM on orthodontic root resorption and the second objective was to assess orthodontic pain.

## Materials and methods

### Trial design and any changes after trial commencement

This was a single-centre, split-mouth trial, with randomization of 1:1 right and left sides as experimental and control/sham. No changes to the methods occurred after trial commencement.

### Participants, eligibility criteria, and settings

The sample consisted of 20 patients that were screened and selected by the authors (except PP) from the orthodontic waiting list of the Department of Orthodontics, The University of Sydney located at Sydney Dental Hospital. All patients required bilateral maxillary first premolar extractions and moderate anchorage. Other inclusion criteria were as follows:

- Permanent dentition;
- Maxillary first premolars with closed apices;
- Similar degree of minimal crowding on each side of the maxillary arch;
- No previous orthodontic or orthopaedic treatment;
- No craniofacial anomalies;
- No history of trauma, bruxism, or parafunction;
- No past or present signs or symptoms of periodontal disease;
- No significant medical history that would affect the development or structure of the teeth and jaws and any subsequent tooth movement.

Informed consent was obtained from the patients and/or parents/caregivers. Ethics approval was obtained from the SLHD Ethics Review Committee (approval numbers X16-0215 and HREC/16/RPAH/266). Patient recruitment commenced in September 2016 and ended in April 2017.

### Intervention

#### Appliance design

**Figure 1.**
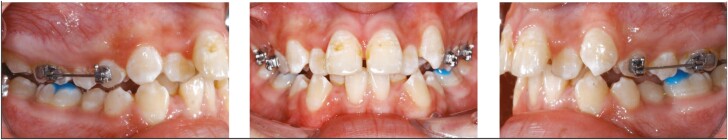
Orthodontic setup.

Self-ligating 0.022-in SPEED brackets were bonded on maxillary first premolars and molars (Strite Industries, Cambridge, Ontario, Canada; Figure 1). Buccal tipping force of 150 g was applied to each maxillary first premolar using a 0.0175 × 0.025 Beta III titanium (3M Unitek, Monrovia, California, USA) cantilever spring which connected the maxillary first permanent molars and first premolars. Occlusal stops (Transbond Plus Light Cure Band Adhesive, 3M Unitek) were bonded to the occlusal surface of the maxillary first molars to disocclude and allow unobstructed tipping of the premolars. The force produced by the springs was calibrated to the nearest gram with a strain gauge (Dentaurum, Ispringen, Germany). All appliances were delivered by the same operator (JS).

#### The OrthoPulse™ device

The OrthoPulse™ device is a compact intraoral PBM device which produces near-infrared light with a continuous 850 nm wavelength from approximately 54 LEDs spaced 5 mm apart in two banks of 3 × 9 elements embedded within a flexible silicone matrix. The LED array is specified to produce a power density of 60 mW/cm^2^.

### Randomization

**Figure 2.**
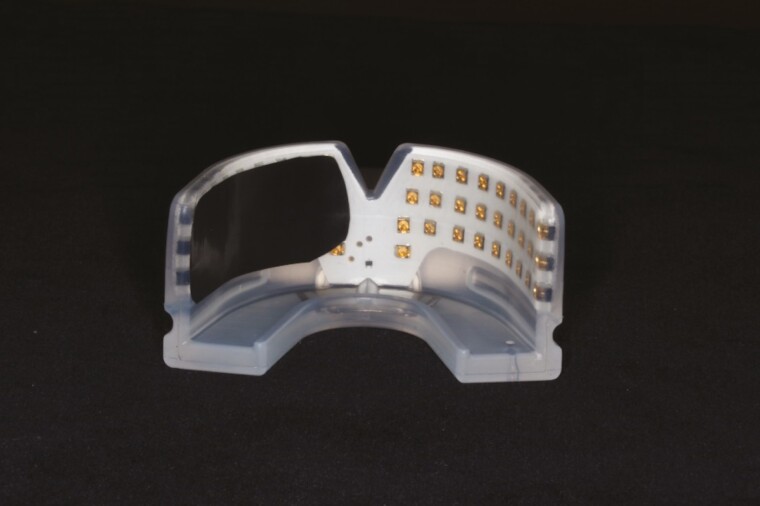
OrthoPulse LED device (left side deactivated/blocked). LED, light-emitting diode.

Using a split-mouth design, each patient was randomly assigned to have the left or right side of their OrthoPulse™ device (Biolux Research, Vancouver, Canada) deactivated to serve as control/sham side, while the contralateral half was the active, experimental side, throughout the experimental period (Figure 2). The right and left sides were randomized to either group using a computerized random number generator (www.randomization.com).

The deactivation was done on each of the LED devices by physically blocking the light emitted from either the left or right banks of LEDs using a single layer of opaque black obstructive film carefully shaped and adapted to the flexible transparent silicone matrix overlying the LEDs (Figure 2). This blocking film was subsequently sealed over with a single layer of medical-grade adhesive tape (Leukoflex, BSN medical GmbH Hamburg, Germany). The light blocking effect of this material was tested with the use of a laser-check sensor (Lasercheck®, Edmund Optics Inc., Barrington, New Jersey, USA) which confirmed complete blockade of the light emitted on the deactivated side.

The patients were then randomly issued the LED devices with either the right or left sides deactivated and received information on proper use as per the manufacturers recommended instructions regarding care, storage, charging, and use. Patients were instructed to use the device for 5 minutes/day on the maxillary arch only, for a total of 28 consecutive days. Compliance was monitored using a smart-phone app which logged each patient’s use of the device via blue-tooth.

### Specimen collection

After the 28-day experimental period the patients had the sectional orthodontic appliances removed, both upper first premolar teeth were carefully extracted to minimize any iatrogenic root damage, under local anaesthesia by a single operator (JS) and the visual analogue scale (VAS) pain self-assessment forms were collected. Extracted teeth were stored in deionized water (Milli Q, Millipore, Bedford, Massachusetts). In preparation for Micro-CT analysis, the premolar teeth were placed in an ultrasonic bath for 10 minutes to allow removal of debris and residual periodontium was removed with damp gauze. Teeth were disinfected by placing them into 70% isopropyl alcohol for 30 minutes, followed by bench drying at room temperature for 48 hours prior to being analysed.

### Outcomes and sample analysis

The primary outcome was the assessment of the effect of LED PBM on orthodontically induced root resorption and the secondary outcome was to evaluate whether application of LED PBM effects the pain perception of patients.

For the evaluation of root resorption crater volumes, teeth were individually scanned using the SkyScan 1172 desktop X-ray machine (SkyScan, Aartselaar, Belgium), using the image resolution set to 17.6 µm.

**Figure 3.**
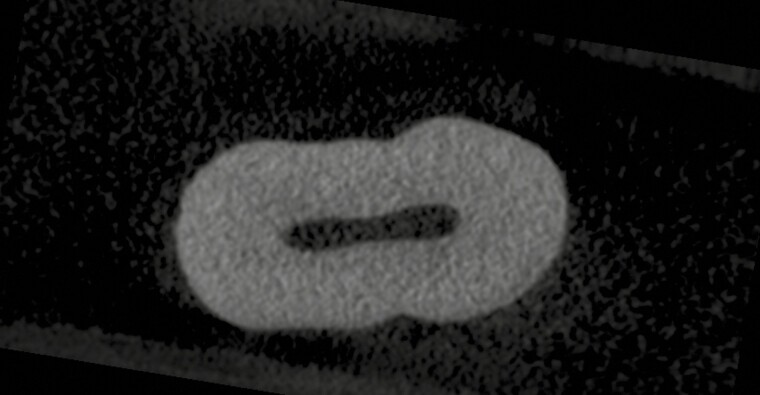
Axial root slice image.

The image data were then subsequently reconstructed to axial two-dimensional slices using software package N-Recon (version 1.6.9.18, Skyscan Aartselaar, Belgium), and the root resorption craters were assessed by examination of axial image stacks of each tooth root (Figure 3). The reconstructed slices were saved in 16-bit tagged image file (TIFF) format. Identification and quantification of resorption craters were performed using the imaging software program Fiji (Version: 2.0.0-rc-15/1.49k, http://imagej.net/Contributors) with a customized macro called Enigma (Australian Centre for Microscopy and Microanalysis, University of Sydney).

All axial slices from each specimen were manually examined for root resorption craters from the cemento-enamel junction to the root apex. Once a crater was detected, it was outlined using a clipping tool and duplicated into its own image sequence. The Enigma macro was then used to determine the volume of each crater in pixels and cubic millimetres. All measurements were carried out by one operator (JS) to reduce bias and error. Craters were categorized and recorded by its location on the root surface (mesial, distal, buccal, and palatal) and by vertical thirds (cervical, middle, and apical). The total root resorption volume for each specimen was also calculated and recorded.

Patients were issued a standardized self-assessment VAS questionnaire to record the amount of orthodontic pain they experienced at 24 hours, 48 hours, 72 hours, and 7 days following the placement of the springs.

### Interim analysis and stopping guidelines

Not applicable.

### Blinding and allocation concealment

Blinding was not possible during the experiment as subjects and the operator could easily identify the deactivated side of their allocated device due to the presence of the blocking film, however blinding was performed for the investigator performing the root resorption crater identification and volume measurements as the teeth were stored in coded vials with no identifying information. Allocation concealment was performed by a staff member not directly involved with the trial. Sealed and opaque envelopes with each patient’s allocation were held in a central location in the department until intervention.

### Sample size calculation

Sample size calculation follows a previously published RCT with the same study design, testing the effect of LLLT on orthodontic root resorption repair based on Ng et al LLLT study on orthodontic root resorption (18, 19). The sample size calculation was carried out at a significance level of 0.05, power of 0.85, the minimum difference of interest of 0.15 mm^3^ and a standard deviation of 0.21 mm^3^, requiring 20 patients.

### Statistical analysis

IBM SPSS Statistics software version 23 (IBM Corporation, USA) was used for the statistical analysis. Paired *t*-tests were performed to determine statistical significance for differences in root resorption and orthodontic pain between the experimental and sham/control sides. The analysis compared crater volumes 1. per tooth, 2. per vertical third, and 3. by surface (mesial, distal, buccal, and palatal).

The total resorption crater volumes of five randomly selected teeth were re-measured to assess intra-operator measurement error and provide a coefficient of variation (CV%).

## Results

### Participant flow and recruitment

**Figure 4.**
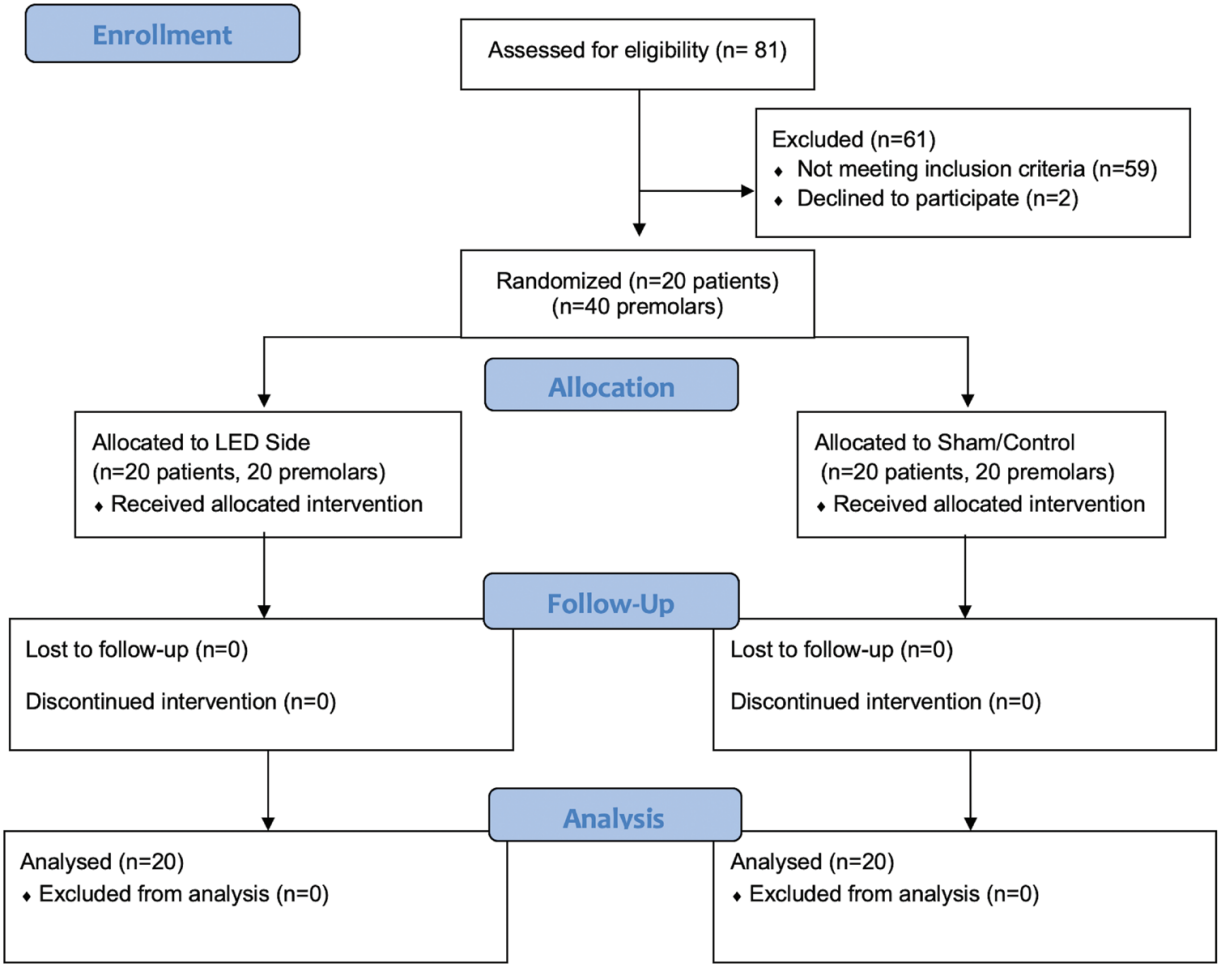
CONSORT flow chart showing patient flow during trial.

All 20 patients completed the study with all 40 premolars eligible for measurement and inclusion in the study as shown with the CONSORT flow diagram in Figure 4. A few patients missed using the device an average of one to two nights and compliance was over 95%.

### Baseline data

Baseline age and sex of the sample are displayed in Table 1. There were 3 male and 17 female patients with a mean age of 15 years 6 months (range, 11 years 5 months to 21 years 5 months).

**Table 1.** Sample baseline characteristics

Sample size (*n*)	Sex	Age, years (SD)
20	3M	M: 16.7 ± 1.2
	17F	F: 15.4 ± 2.8

### Primary and secondary outcomes

#### Root resorption crater volumes

**Figure 5.**
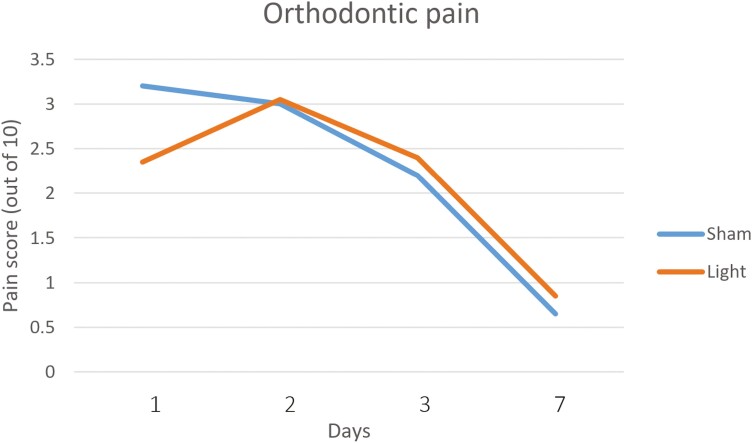
Orthodontic pain over time.

In general, PBM was not associated with a significant difference in root resorption when compared with sham/control side (Figures 5 and Tables 2–4). In terms of total volume of root resorption, PBM-treated premolars averaged 0.216 mm^3^ while sham/control premolars averaged 0.284 mm^3^ (Table 2 and Figure 5). Thus, PBM resulted in 0.068 mm^3^ or 24% less root resorption compared with regular orthodontic tooth movement. However, this difference was not statistically significant (*P* = 0.308). Twelve of the 20 subjects exhibited more total root resorption on the deactivated side when compared with LED (Table 2). When categorized into tooth surfaces, only the distal surfaces exhibited more mean root resorption on the PBM side, however this was also not statistically significant (*P* = 0.477), Table 3.

**Table 2.** Total root resorption values per tooth

Subject	Control (mm^3^)	LED RR (mm^3^)	Difference
1	0.132	0.157	0.025
2	0.077	0.018	−0.060
3	0.167	0.245	0.078
4	0.044	0.088	0.045
5	0.858	0.139	−0.719
6	0.199	0.081	−0.118
7	0.058	0.026	−0.032
8	0.395	0.213	−0.182
9	0.157	0.041	−0.116
10	0.066	0.212	0.146
11	0.751	0.441	−0.310
12	0.189	0.242	0.053
13	0.145	0.115	−0.030
14	0.349	1.055	0.706
15	0.774	0.099	−0.675
16	0.416	0.321	−0.095
17	0.566	0.382	−0.185
18	0.109	0.055	−0.054
19	0.119	0.150	0.032
20	0.114	0.233	0.119
Total	5.686	4.313	−1.373
Mean	0.284	0.216	−0.069
Std Dev	0.259	0.229	0.293
*P*			0.308
95% CI			−0.20586 to 0.069

CI, confidence interval; LED, light-emitting diode.

**Table 3.** Root resorption per tooth surface

Tooth surface	LED			Control			Difference			
	Total	Mean	SD	Total	Mean	SD	Mean Diff	95% CI		*P*
								Lower bound	Upper bound	
Buccal	1.174	0.059	0.056	1.929	0.096	0.121	−0.038	−0.098	0.022	0.203
Palatal	0.288	0.014	0.019	0.914	0.046	0.091	−0.031	−0.075	0.012	0.148
Mesial	1.200	0.060	0.082	1.736	0.087	0.173	−0.027	−0.100	0.047	0.455
Distal	1.647	0.082	0.199	1.106	0.055	0.070	0.027	−0.051	0.105	0.477

CI, confidence interval; LED, light-emitting diode.

**Table 4.** Root resorption per vertical third

Tooth surface	LED			Control			Difference			
	Total	Mean	SD	Total	Mean	SD	Mean Diff	95% CI		*P*
								Lower bound	Upper bound	
Coronal	1.864	0.093	0.097	2.438	0.122	0.155	−0.029	−0.109	0.051	0.461
Middle	1.730	0.086	0.135	1.472	0.074	0.176	−0.013	−0.105	0.079	0.772
Apical	1.518	0.076	0.138	0.977	0.049	0.063	−0.027	−0.098	0.044	0.437

Repeat measurements on five randomly selected premolars resulted in an overall standard error (root mean square error) of 0.007 and a coefficient of variation of 1.8%.

#### Orthodontic pain

The mean pain scores were significantly lower on the PBM side 24 hours after the placement of the springs (*P* = 0.023, Table 5). On subsequent time points (48 hours, 72 hours, and 7 days) the mean pain scores were only marginally higher on the PBM side compared with the deactivated side, although these differences were not statistically significant (*P* = 0.924, 0.479, and 0.214, respectively, Table 5).

**Table 5.** VAS pain scores (out of 10)

Hours/days	Mean	SD	Mean	SD	Mean Diff	95% CI		*P*
						Lower bound	Upper bound	
24 h	2.35	1.725	3.2	2.02	−0.85	−1.57	−0.13	0.023
48 h	3.05	1.932	3	1.97	0.05	−1.03	1.13	0.924
72 h	2.4	1.984	2.2	1.47	0.2	−0.38	0.78	0.479
7 days	0.85	1.461	0.65	1.09	0.2	−0.13	0.53	0.214

CI, confidence interval; VAS, visual analogue scale.

#### Harms

No harms were observed during the experimental period and the self-administration of LED illumination was entirely without complication.

## Discussion

This split-mouth prospective pilot RCT evaluating the effect of intraoral LED-mediated PBM on OIIRR showed that although the side receiving LED PBM had minimally less total root resorption, this was not statistically significant.

### LLLT and LED PBM

The precise biological mechanism which underpins the therapeutic effects of LLLT and LED PBM are not yet fully understood. These effects are thought to be dependent upon an increase in blood supply via vasodilation due to the increased production of nitric oxide and other pro-inflammatory mediators such as IL-1b, PGE1, increased ATP production by the mitochondria and increased cellular metabolism (20, 21). There is still debate over which light source should be used for best therapeutic outcomes as it was initially believed that LLLT or LED PBM required the use of coherent laser light. Lasers are coherent spatially and temporally and the light is focussed, however are more expensive and require application of a trained specialist (5, 20). Several animal and human studies were done investigating the effects of LLLT on orthodontic tooth movement and root resorption. Animal experiments showed that LLLT significantly increased the osteoblast and fibroblast numbers and increased cementum thickness (22, 23). While two human studies showed no detrimental effects of LLLT on orthodontic root resorption during canine retraction (10, 12) another RCT showed that the side receiving LLLT showed 23% less orthodontic root resorption (18).

Although the effects of LLLT on orthodontic tooth movement and root resorption appear promising, the need for additional equipment for the clinician and associated costs together with the need for patients to attend more frequent appointments present challenges in terms of the cost–benefit proposition (3). LED is a lower cost alternative to LLLT and it is also considered safer for home application since the incoherent light from LED has less potential to harm human tissues. There are only a limited number of studies investigating the effects of LED phototherapy on orthodontic root resorption (8, 14–17). Fonseca *et al.* (15) studied LED phototherapy and orthodontic root resorption in Wistar rats over a 7-day experimental period and concluded that LED therapy improved periodontal tissue repair and reduced the amount of inflammation and root resorption after the application of orthodontic force. Nimeri *et al.*’s (8) study on the effect of extraoral LED PBM on root resorption in orthodontic patients concluded that LED light did not cause root resorption greater than the normal range commonly seen with conventional treatment. However, this study included patients with a wide range of crowding and different orthodontic treatment durations and procedures, i.e. alignment or space closure, with no control group comparison. Root resorption metrics were assessed by comparing root lengths on CBCT before and after alignment in both growing and non-growing patients. Another recent study evaluated the effects of LED modulated PBM and LLLT on orthodontic root resorption compared with a placebo group, with no significant differences found with either application (17). However, this study also had limitations such as small sample size and not using a placebo LED PBM group.

Although these previous studies had various methodological shortcomings, their results are in agreement with our study in that no statistically significant difference was found in the amount of root resorption between the experimental and control sides. Since PBM has been shown to improve healing and reduce pain and inflammation (13), it was expected that less root resorption would be seen following the application of buccal tipping forces over a 28-day period, in line with the findings of a previous RCT using LLLT and the same study design (18). This difference could be due to a few factors that may affect the outcomes of light therapy in general. The effects of PBM are versatile, and depending on the light parameters used, it can elicit both stimulatory and inhibitory responses (24). The basic Arndt–Schultz law states that ‘small doses stimulate living systems, medium doses impede, and large doses destroy’ (25). In addition to this, very small doses fail to stimulate. The energy density/dose dependency of PBM effects has been demonstrated in several studies (26). Furthermore, the level of penetration of the light source into tissues and reaching the periodontium is affected by several factors including tissue thickness, pigmentation, keratinization, hydration, maturity, and the depth of the roots within the alveolar housing (27). This poses challenges in the search for the optimal light parameters to achieve tooth movement acceleration while limiting root resorption, as there is likely to be a high degree of variability in the amount of light energy loss and tissue penetration between patients with different soft and hard tissue characteristics, and this needs to be adjusted for. An animal study comparing the effects of LLLT and LED on orthodontic tooth movement and root resorption found that neither method accelerated tooth movement, LED therapy had no significant effect on root resorption, and LLLT increased root resorption (28). These findings were attributed to the differences in characteristics of both light sources and the amount of light penetration achieved.

### Effects of LED PBM on orthodontic pain

The literature reports two main mechanisms responsible for the analgesic effect of PBM. The first is an immediate direct effect on nerve fibre conduction by stabilizing membrane potentials which could reduce the propensity for depolarization (neural blockade). The second is via effects on the biochemical inflammatory process including the release of beta-endorphins (29–31). This study is the first split-mouth RCT utilizing LED PBM for orthodontic pain following orthodontic adjustment. Our results showed significantly less orthodontic pain on the LED side at only the 24-hour time point, although the difference between both sides was small. The onset of orthodontic pain following various types of orthodontic force application is normally around 2–4 hours, peaking at 24–36 hours, with a gradual decrease after 48 hours, reaching a minimum at day 7 (32, 33). LLLT studies have demonstrated overall reduction in pain intensity, later onset of pain and earlier resolution of pain following various applications of orthodontic force including initial archwire placement, separator placement, and canine retraction (11, 34, 35). In our study, the LED exposed side exhibited peak pain intensity at day 2, and although the intensity was subjectively reported to be lower in comparison to the control side peak, this difference did not reach statistical significance. There are two studies utilizing LED PBM for pain management following placement of separators (32, 36). Figueira *et al.* showed that LED application via a probe significantly reduced pain at 48 hours, 72 hours, and 1 week (32), however this was not a split-mouth study. Esper *et al.* compared the effects of LED with LLLT on a group of volunteers that served first in the placebo and then the experimental groups (36). Similar to our study, their LED group experienced the peak pain at 48 hours, but their pain scores were significantly lower at all time points compared with the controls. These disparities could be due to the differences in several methodological factors such as the orthodontic force application method, as well as the light parameters and absorption. This is similar to the variable effects of PBM on tooth movement and root resorption and could be responsible for the diminishing analgesic effect of LED light after the initial 24 hours, when the concentrations of local inflammatory mediators approach their peak.

### Limitations

The inability to perform blinding is one of the limitations of this study. As the method used to deactivate one side of the LED device involved the use of a clearly visible barrier, the operator, and the subjects were aware of which side was active. Nonetheless, most patients reported that they perceived a warm and pleasant sensation on the illuminated side which brings into question the possibility of blinding patients to LED-mediated PBM at all. This inevitably influences the validity of having a sham side. The force level chosen in this study is considered to be a moderate force level. It may be suggested that lighter forces are needed to initiate orthodontic tooth movement, however we chose a force level that was previously shown to create root resorption craters, while still not being too high. The relatively short 28-day duration of the study is also a limitation as it does not reflect the true clinical situation where orthodontic treatment extends over many months to years. The compliance of patients was not 100% during the experimental period in using the PBM device with some patients missing one to two nights. Considering the short experimental period, this is a limitation of the study however this is also reflective of real life, as patients are not 100% compliant with most orthodontic treatment necessities (37). A possible crossover effect using the split-mouth study design could be considered another limitation of this study. However, since LED light intensity decreases significantly upon entry into tissues, a crossover effect is unlikely (27, 38). This still needs to be investigated in future studies.

### Generalizability

The results of this study can only be generalizable when using the same LED PBM protocol and with the application of initial forces that can generate orthodontic root resorption. Our study was conducted over a 28-day period for ethical reasons, and this also affects the generalizability of our results. The buccal tipping force used in this study was chosen as it provides a clinically acceptable force that is still able to stimulate the root resorption process, using standard orthodontic techniques. Resorption craters formed at this force level over the 28-day experimental period have proven to be detectible in numerous other root resorption studies (18, 39). As this study did not show statistically significant differences between the groups, it may be reasonable to follow subjects over a longer experimental period to allow the proposed effects to manifest and develop over time with different types of forces acting upon the dentition, in addition to different PBM protocols.

## Conclusion

This 28-day pilot randomized split-mouth controlled trial showed that using OrthoPulse for 5 minutes/day for the application of LED-mediated PBM is not associated with any difference in orthodontic root resorption when the maxillary first premolars are subjected to a 150 g buccal tipping force. This LED-mediated PBM application is associated with marginally lower orthodontic pain 24 hours after the application of orthodontic tipping force, but not after 48 hours, 72 hours, or 7 days. Future studies should be performed on patients undergoing a full course of orthodontic treatment which may be more representative of clinical practice.

## Data Availability

The datasets used and/or analysed during the current study are available from the corresponding author on reasonable request.
